# Coagulating Colubrids: Evolutionary, Pathophysiological and Biodiscovery Implications of Venom Variations between Boomslang (*Dispholidus typus*) and Twig Snake (*Thelotornis mossambicanus*)

**DOI:** 10.3390/toxins9050171

**Published:** 2017-05-19

**Authors:** Jordan Debono, James Dobson, Nicholas R. Casewell, Anthony Romilio, Bin Li, Nyoman Kurniawan, Karine Mardon, Vera Weisbecker, Amanda Nouwens, Hang Fai Kwok, Bryan G. Fry

**Affiliations:** 1 Venom Evolution Lab, School of Biological Sciences, University of Queensland, St. Lucia, QLD 4072, Australia; jordan.debono@uqconnect.edu.au (J.D.); j.dobson@uq.edu.au (J.D.); 2 Alistair Reid Venom Research Unit, Parasitology Department, Liverpool School of Tropical Medicine, Pembroke Place, Liverpool L3 5QA, UK; nicholas.casewell@lstmed.ac.uk; 3 Vertebrate Palaeontology and Biomechanics Laboratory, School of Biological Sciences, The University of Queensland, St. Lucia, QLD 4072, Australia; a.romilio@uq.edu.au (A.R.); v.weisbecker@uq.edu.au (V.W.); 4 Faculty of Health Sciences, University of Macau, Avenida da Universidade, Taipa, Macau SAR; yb47627@umac.mo (B.L.); hfkwok@umac.mo (H.F.K.); 5 Centre for Advanced Imaging, University of Queensland, St. Lucia, QLD 4072, Australia; nyoman.kurniawan@cai.uq.edu.au (N.K.); k.mardon@uq.edu.au (K.M.); 6 School of Chemistry and Molecular Biosciences, University of Queensland, St. Lucia, QLD 4072, Australia; a.nouwens@uq.edu.au

**Keywords:** procoagulant, boomslang, *Dispholidus typus*, *Thelotornis mossambicanus*, colubridae, enzyme activity, biodiscovery, evolution

## Abstract

Venoms can deleteriously affect any physiological system reachable by the bloodstream, including directly interfering with the coagulation cascade. Such coagulopathic toxins may be anticoagulants or procoagulants. Snake venoms are unique in their use of procoagulant toxins for predatory purposes. The boomslang (*Dispholidus typus*) and the twig snakes (*Thelotornis* species) are iconic African snakes belonging to the family Colubridae. Both species produce strikingly similar lethal procoagulant pathologies. Despite these similarities, antivenom is only produced for treating bites by *D. typus*, and the mechanisms of action of both venoms have been understudied. In this study, we investigated the venom of *D. typus* and *T. mossambicanus* utilising a range of proteomic and bioactivity approaches, including determining the procoagulant properties of both venoms in relation to the human coagulation pathways. In doing so, we developed a novel procoagulant assay, utilising a Stago STA-R Max analyser, to accurately detect real time clotting in plasma at varying concentrations of venom. This approach was used to assess the clotting capabilities of the two venoms both with and without calcium and phospholipid co-factors. We found that *T. mossambicanus* produced a significantly stronger coagulation response compared to *D. typus*. Functional enzyme assays showed that *T. mossambicanus* also exhibited a higher metalloprotease and phospholipase activity but had a much lower serine protease activity relative to *D. typus* venom. The neutralising capability of the available boomslang antivenom was also investigated on both species, with it being 11.3 times more effective upon *D. typus* venom than *T. mossambicanus*. In addition to being a faster clotting venom, *T. mossambicanus* was revealed to be a much more complex venom composition than *D. typus*. This is consistent with patterns seen for other snakes with venom complexity linked to dietary complexity. Consistent with the external morphological differences in head shape between the two species, CT and MRI analyses revealed significant internal structural differences in skull architecture and venom gland anatomy. This study increases our understanding of not only the biodiscovery potential of these medically important species but also increases our knowledge of the pathological relationship between venom and the human coagulation cascade.

## 1. Introduction

Venoms are used for competitor deterrence, defence, and prey capture. These chemical cocktails use myriad protein types as starting substrates for the evolution of their toxic arsenals. Many of these protein families have been convergently recruited into the secretions of various venomous animal classes [1]. Novel expression of a normal body protein in the venom gland is followed by fixation and diversification of the proteins, with duplication and diversification leading to neofunctionalisation. In reptiles, such compounds have been recruited for use at different times in the diverse history of venom in this lineage with relative expression levels rising and falling for a particular toxin type along the evolutionary continuum [2]. As venoms evolve through the birth-and-death model of protein evolution [3], there are many instances of toxins also being lost from within a lineage [4].

Rather than prey death, prey immobilization is the driving selection pressure upon the venom, as there is no functional difference between a prey item that is physically unable to flee or defend itself and one that is dead [5,6]. Prey capture involving chemicals will produce a different form of injury than one involving mechanical subjugation techniques such as claws or cutting teeth. Interactions among toxins are selected for a combined function, which facilitates prey subjugation in a time dependent manner. Evolutionary pressures from other variables such as post-envenomation prey detectability and prey escape potential also shape venoms. The evolutionary pressures acting upon predatory venoms select specific actions that facilitate prey capture via target sites reachable by the blood. Actions upon nerve action potentials and the blood clotting cascade are two particularly important areas. Venoms that act on the nerves are typically used by species that include reptiles as major prey items in their diet. The specific function selected for the case of elapid snakes, which feed on sleeping lizards, is caused by alpha-neurotoxins, which antagonistically bind to the post-synaptic nicotinic acetylcholine receptor to produce flaccid paralysis [7]. Conversely, the long-glanded blue coral snake (*Calliophis bivirgatus*) and mambas (*Dendroaspis* species), which feed on active prey items with a high escape potential, have presynaptic-acting venoms that produce excitatory neurotoxicity, resulting in uncontrollable, uncoordinated spastic paralysis [6]. In the case of *C. bivirgatus*, there is an additional selection pressure for rapid acting venom due to specialisation upon other venomous snakes, which in turn have a high chance of prey retaliation.

Toxins that target the blood coagulation cascade do so in one of two mutually exclusive functional mechanisms; overall net anticoagulation or procoagulation. Anticoagulant venoms, such as those found in most pit-vipers, participate in the formation of haemorrhagic shock through a combined chemical assault, which may include the use of procoagulant pathways as part of the net anticoagulant function. Targets ranging from the cleavage of fibrinogen (whether outright destruction or the use of procoagulant pathways to produce weak, unnatural clots that are readily broken down by the abundant plasmin produced by synergistic toxins, which activate plasminogen) through to platelets (aggregation inhibition or unnatural clumping effects, which disrupt the normal role platelets serve in clotting), with these toxic effects accompanied by other toxins that increase the permeability of the vascular bed [1,2,8,9,10,11,12]. These venoms are often employed by ambush feeding vipers, which specialise upon warm-blooded prey items, and are able to be efficiently tracked post-bite using chemoreception and heat detection sensory arrays [13,14]. In contrast, strong clot forming procoagulant venoms are selected for lineages that feed upon prey with fast moving blood circulation rates. Such prey lineages are vulnerable to toxins that produce blood clots that rapidly subjugate through stroke induction, such as taipans (*Oxyuranus* species) feeding upon rodents [15].

The African boomslang (*Dispholidus typus*) and the twig snakes (*Thelotornis* species) are closely related iconic African snakes belonging to the family Colubridae, and both inhabit arboreal, predatory niches that include prey items with a high potential for escape [16,17]. The limited work that has been undertaken upon them has revealed that both have clot-forming, procoagulant venoms [17,18,19,20,21,22]. While the venoms appear to be functionally similar in their potent action upon blood chemistry, the relative complexity of the venoms is unknown. While *Dispholidus* has been shown to have a venom dominated by P-III snake venom metalloprotease (SVMP) [22,23,24], other toxin types are present in the venom in lower amounts [3,23]. In vipers, certain procoagulant P-III SVMPs have been shown to activate prothrombin [25,26,27,28] or Factor X [27,28,29]. Strong clot forming procoagulant function has convergently evolved in the venom of *D. typus* and similar activities are suggested for *T. capensis* [30]. The venom composition for any *Thelotornis* species is unknown so the toxin type responsible for procoagulation can only be inferred as likely being a P-III SVMP like its related genera *Dispholidus*. From a functional perspective, these two related but very divergent snakes share a coagulotoxic action with their last common ancestor [3,23]. These snakes have evolved procoagulant venoms convergently in relation to vipers in the *Daboia* genus [31,32] or elapids in the *Oxyuranus* or *Pseudonaja* genera [33,34]. *Daboia* use serine proteases to activate factor X, while *Oxyuranus* and *Pseudonaja* use a mutated factor Xa:factor Va complex to escape hemostatic control [35]. 

*Dispholidus* is genetically close to the genus *Thrasops*. *Dispholidus* and *Thrasops* are in turn sister to *Thelotornis*. The two genera studied here differ in their occupied niches and morphology. *Dispholidus* is a monotypic genus consisting of the boomslang (*D. typus*), which is an active foraging snake with very large eyes with circular pupils and acute vision, a large round head, and variable colour, ranging from uniform brown to green with darkened scale edges [17,20]. The large head is quite similar to the tree cobras (e.g., *Naja goldii*), including enlarged eyes. In contrast, *Thelotornis* is a genus of ambush feeding, highly cryptically and intricately patterned snakes with large eyes, horizontal pupils, and near binocular vision [17,20,36]. Their elongate heads are convergently similar to other Colubridae, particularly the *Ahaetulla* genus from Asia and the *Oxybelis* genus of the southern U.S. and Central America. 

Birds account for the majority of *Dispholidus* prey items as it hunts in the canopy. The fast-moving, endothermic blood circulatory system of avians is similar to mammals in being vulnerable to rapid prey subjugation by stroke formation, thus neutralising the high escape potential posed by prey items with flight ability. While lizards such as chameleons form a percentage of the *Dispholidus* diet [16,17], they are non-dangerous and slow moving and are also taken during their diurnal activity period when blood circulation is at its maximum. *Thelotornis* may have a more complex diet than *D. typus* as it occupies a niche located near or on the forest floor and snakes of this genus are perhaps also more likely to be sensitive to prey escape potentials due to their slower movements relative to the fast moving *Dispholidus* [16,17,36]. 

Neither genus of snakes was thought to be of significant medical consequence until each killed an eminent herpetologist (Robert Mertens and Karl Patterson Schmidt, respectively) [24]. However, the ability for *D. typus* to cause serious harm to humans, and even death, had previously been reported by F.W. Fitzsimons as early as 1909 [17,20,21,37,38,39,40]. Strikingly, *T. capensis* has been shown to produce nearly identical human clinical symptoms as reported for *D. typus*, with death the result of internal bleeding such as cerebral haemorrhage [19,30,41]. However, the existing antivenom manufactured to treat bites caused by *D. typus* is said to be ineffective in cases of *T. capensis* envenomation [17,21,30]. While prey injected with significant concentrations of procoagulant venoms items succumb to stroke [42], human deaths from these venoms are due to internal haemorrhage due to the dilution of the procoagulant venom into a volume of blood much larger than the concentrated effects in prey animals, resulting in defibrinogenation due to venom induced consumptive coagulopathy [2,15,28,41,43].

Despite a monovalent antivenom existing and routinely administered to bite victims [17,22], limited research has been conducted on the function of *D. typus* venom, with the literature being dominated by bite case reports or proteomics in the absence of bioactivity testing. Even less is known about *Thelotornis* venoms, with limited previous publications having mainly dealt only with *T. capensis*, while the rest of the genus is unknown in its venom composition. We aimed to investigate the key similarities and differences between the two genera utilising a range of proteomic and bioactivity approaches to reconstruct their functional evolution in response to prey preference and ecological niche occupied. It is hypothesised that *T. mossambicanus* has a greater diversity of venom toxin components, reflective of its diverse diet and their associated heighted potential for prey escape, than the more specialised feeder *D. typus*.

## 2. Results

### 2.1. Skull and Venom Gland Anatomical Comparisons

**Figure 1:**
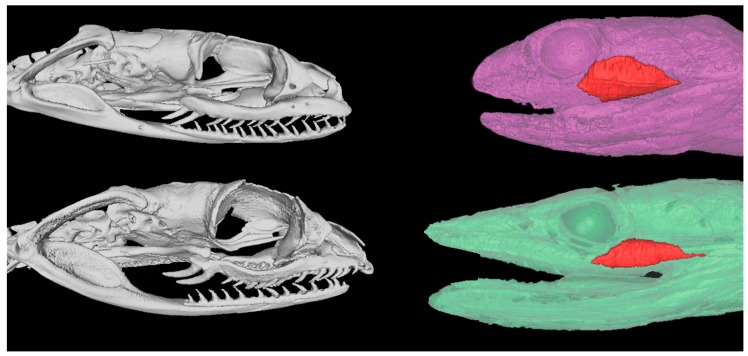
Contrast tomography (left column) and magnetic resonance imaging (right column) comparison of *D. typus* (top row) and *T. mossambicanus* (bottom row) venom delivery systems.

Both species displayed enlarged rear-fangs, consistent with their demonstrated efficient venom delivery in predation and also defensive bites. Reflective of their difference in head shape, the *T. mossambicanus* venom gland was more elongate than that of *D. typus* due to contributions by both hard and soft tissue (Figure 1).

### 2.2. Proteomics 

**Figure 2:**
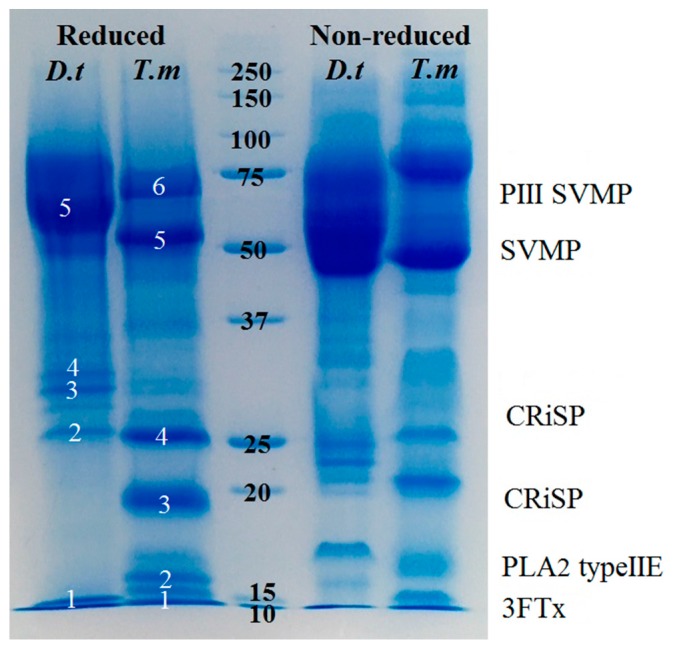
1D SDS PAGE with identified groups of proteins. *D.t* = *Dispholidus typus*, *T.m* = *Thelotornis mossambicanus*. Reduced conditions on the left, Non-reduced conditions on the right. Molecular weight marker indicated by centered numbers (kDa). Annotations (right) refer to bands identified (white numbers) within the reduced column.

**Figure 3:**
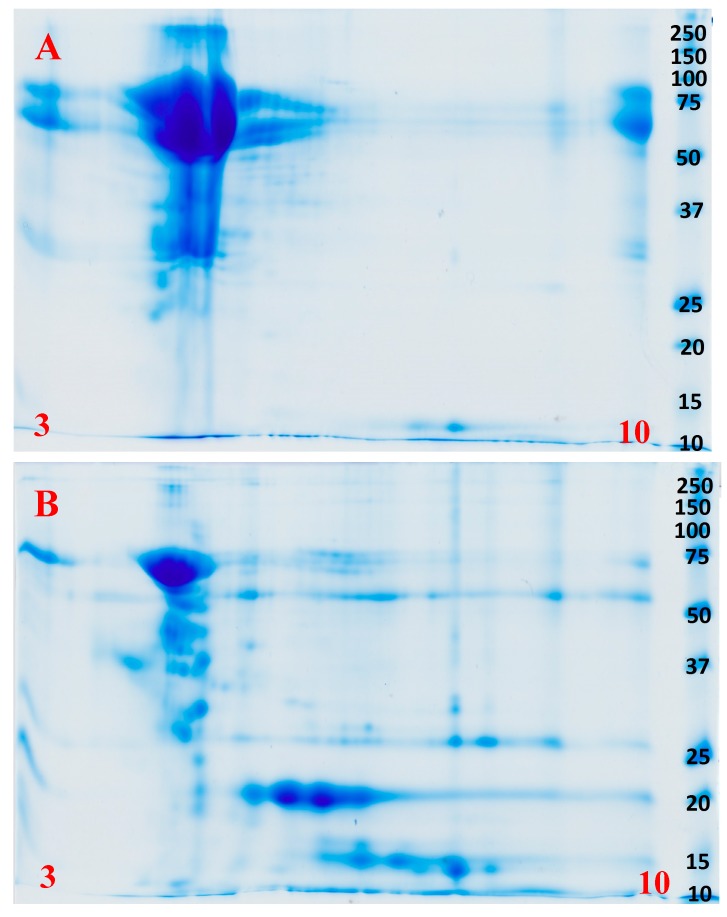
(**A**) 2D SDS PAGE of *Dispholidus typus* under reducing conditions; (**B**) 2D SDS PAGE of *Thelotornis mossambicanus* under reducing conditions. Molecular weight markers (kDa) are as for Figure 1. pI range is 3–10 (left to right) in red.

Shotgun mass spectrometry analysis recovered the same toxin types from each venom; CRiSP, PLA_2_ Type IIE, 3FTx, and SVMP. However, 1D and 2D gels showed significantly different relative abundances and complexity levels. While both 1D and 2D gels indicate that both species are dominated by P-III SVMP (Figure 2 and Figure 3, Supplementary Data), *T. mossambicanus* also contained phospholipase A_2_ (PLA_2_ Type IIE) in high amounts. The presence of PLA_2_ Type IIE is a significant discovery as it is the third type of PLA_2_ to be characterised in snake venoms [44]. The molecular weights of identified P-III SVMP correspond to previous transcriptome and proteome data for *D. typus* [24].

### 2.3. Enzymatic Assays

#### Fluorescent Determination of Matrix Metalloprotease and Kallikrein Activity

**Figure 4:**
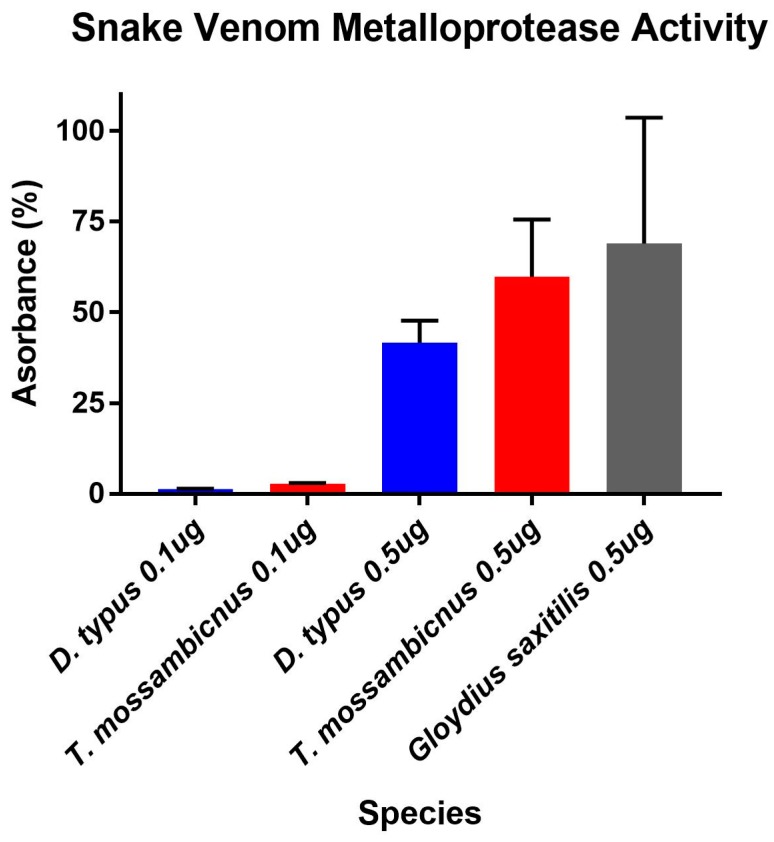
Snake Venom Metalloprotease Activity of venom (10 ng/µL and 50 ng/µL) was measured based on its ability to cleave a fluorogenic peptide substrate (Mca-PLGL-Dpa-AR-NH2 Fluorogenic MMP Substrate, Cat#ES001). Column graph of Matrix Metalloprotease activity assay of *D. typus* and *T. mossambicanus* obtained from normalisation of slope values. X axis: species name and concentration; Y axis: absorbance percentage. Analysis of triplicates was conducted on GraphPad PRISM 7.0 and error bars indicate standard deviation.

Despite *T. mossambicanus* having a more complex venom profile than *D. typus*, and thus lower relative amounts of P-III SVMP (Supplementary Data), it actually displayed a higher rate of cleavage of a matrix metalloprotease specific substrate (Figure 4). A two-way (RM) ANOVA (α = 0.05) indicates that there is a significant difference in Column Factor (species and concentration), accounting for 78.6% of the total variance (after adjusting for matching); *F*(3, 8) = 45.2, *p* < 0.001. Tukey’s post-hoc test indicated that there is a significant difference in activity between *D. typus* and *T. mossambicanus* at a concentration of 0.5 µg/100 µL (*p* ≤ 0.04). The levels of *T. mossambicanus* were of a high level, similar to that of the viperid snake *Gloydius saxitilis* included for comparison.

**Figure 5:**
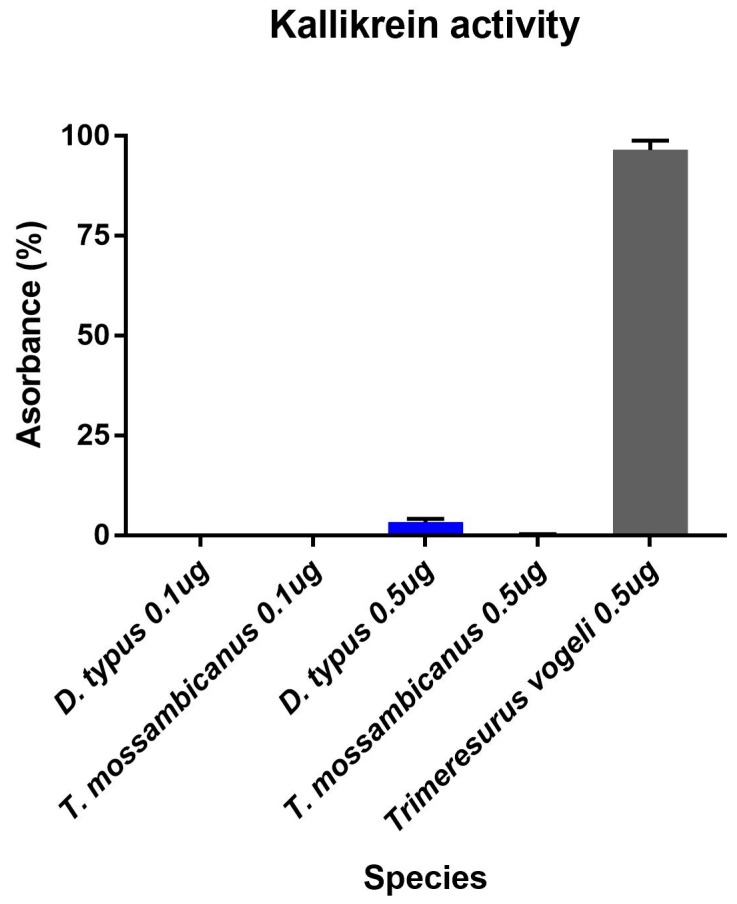
The kallikrein activity of venom (10 ng/µL and 50 ng/µL) was measured based on its ability to cleave a fluorogenic peptide substrate (Boc-VPR-AMC Fluorogenic Peptide Substrate, Cat#ES011). Column graph of Kallikerin activity assay of *D. typus* and *T. mossambicanus* obtained from normalisation of slope values. There is a highly significant difference in activity between *D. typus* and *T. mossambicanus* at both concentrations (*p* ≤ 0.001). X axis: species name and concentration; Y axis: absorbance as a percentage. Analysis of triplicates was conducted on GraphPad PRISM 7.0 and error bars indicate standard deviation.

Contrastingly, *D. typus* venom demonstrated a much higher kallikrein activity compared to that of *T. mossambicanus*. *D. typus* had an active concentration of 0.1 µg/100 µL and 0.5 µg/100 µL, compared to *T. mossambicanus*, which was only slightly active at 0.5 µg/100 µL (Figure 5). A two-way (RM) ANOVA (α = 0.05) indicates that there is a significant difference within the test, with Column Factor (species and concentration) accounting for 80.1% of the total variance (after adjusting for matching); *F*(3, 7) = 136.6, *p* ≤ 0.001. Tukey’s post-hoc test indicated that there is a highly significant difference in activity between *D. typus* and *T. mossambicanus* at both concentrations (*p* ≤ 0.001). This is indicated by the increased presence of kallikrein-like serine proteases in *D. typus*’ venom profile (Figure 2). However, even the proportionally higher *D. typus* activity levels were insignificant in relation to the representative viperid *Trimeresurus vogeli* included for comparison, thus indicating that this function is a trivial aspect of the venom, consistent with the non-detection in our proteomics and low-levels in the published *D. typus* venom gland transcriptome [24].

### 2.4. Fluorescent Determination of sPLA_2_ Activity

**Figure 6:**
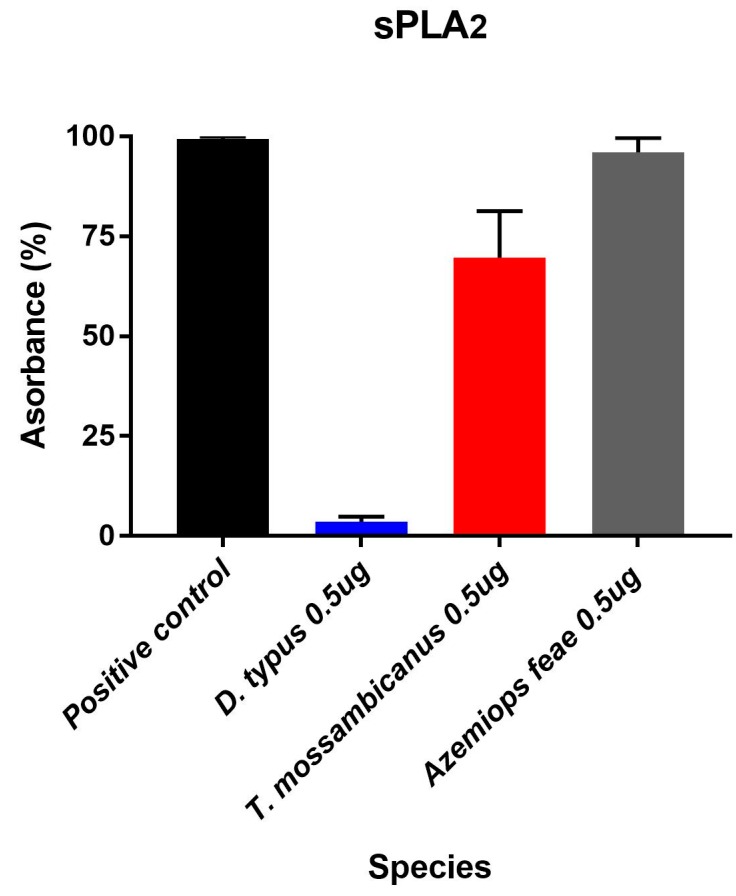
Secretory Phospholipase A_2_ was measured by its ability to cleave a fluorogenic peptide substrate EnzChek^®^ (Cat# E10217). Column graph of sPLA_2_ activity assay of *D. typus* and *T. mossambicanus* obtained from normalisation of slope values. There is a highly significant difference in activity between *D. typus* from the positive control and between *D. typus* and *T. mossambicanus* (*p* ≤ 0.001). There is also a significant difference of *T. mossambicanus* from the positive control (*p* ≤ 0.01). X axis: species name and concentration; Y axis: slope as a relative percentage. Analysis of triplicates was conducted on GraphPad PRISM 7.0 and error bars indicate standard deviation.

We tested for continuous secretory phospholipase A_2_ (sPLA_2_) enzymatic activity over 100 measurement cycles using and following the EnzChek^®^ Phospholipase A_2_ Assay Kit (Cat#E10217). *T. mossambicanus* showed increased levels of sPLA_2_ activity compared to *D. typus* at a concentration of 50 ng/µL (Figure 6). This increase in activity by *T. mossambicanus* is also mimicked in the venom profiling of both venoms via 1D SDS PAGE (Figure 2). A two-way RM ANOVA (α = 0.05) indicates that there is a significant difference within the assay, with Column Factor (species and concentration) accounting for 63.41% of total variance (after adjusting for matching); *F*(2, 6) = 78.75, *p* ≤ 0.001. Tukey’s post-hoc test indicated there is a highly significant difference in activity between D. typus from the positive control and between *D. typus* and *T. mossambicanus* (*p* ≤ 0.001). Tukey’s post-hoc test also indicates a significant difference of *T. mossambicanus* from the positive control (*p* ≤ 0.01). The *T. mossambicanus* levels were similar to that of the representative viperid snake *Azemiops feae* included for comparison.

### 2.5. Procoagulation Analysis

**Figure 7:**
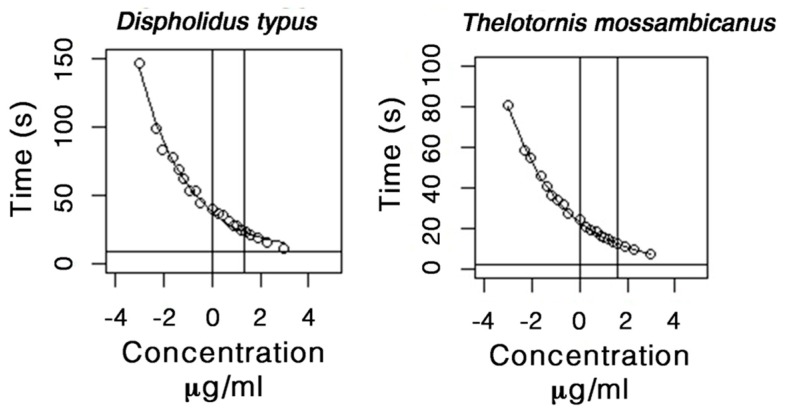
Asymptotic plots of *D. typus* and *T. mossambicanus* procoagulation clotting against human plasma. Asymptotic time depicted by horizontal line, *D. typus* asymptote 9.09 (s), *T. mossambicanus* asymptote 2.06 (s). X axis: log concentration (µg/mL). Y axis: Time in seconds. Analysis and plots created in R Studio.

**Figure 8:**
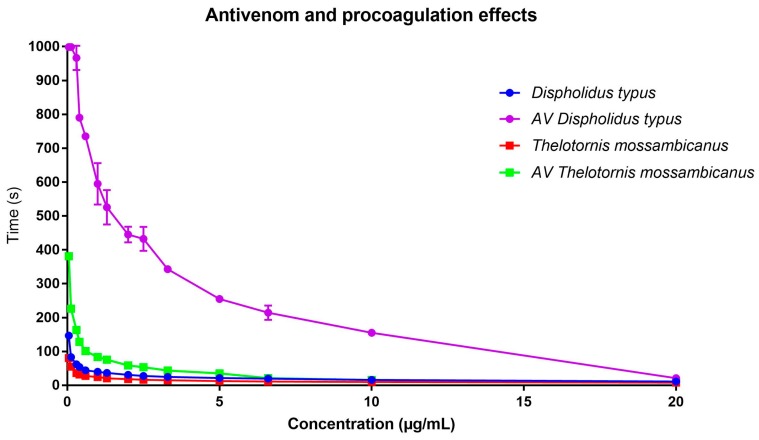
Comparison of procoagulation clotting curves of *D. typus* and *T. mossambicanus*, with and without the addition of antivenom. *D. typus* clotting curve given in blue circles, AV *D. typus* = antivenom response curve given in purple circles, *T. mossambicanus* clotting curve given in red squares, AV *T. mossambicanus* = antivenom response curve given in green squares. X axis: final venom concentration (µg/mL), Y axis: clotting time in seconds. Values are averages of triplicates (single dilution measured three times) and standard deviation error bars are shown for each, although for most the error range is smaller than the line icon.

While both venoms were potently procoagulant, we find that the venom of *T. mossambicanus* coagulates plasma much faster that that of *D. typus* (Figure 7 and Figure 8). At a 20 µg/mL concentration, *T. mossambicanus* venom clots the plasma on average in 7.5 s (SD 0.15 s), compared to *D. typus*, which clots the plasma on average in 11.2 s (SD 0.06 s) (Table 1). The *T. mossambicanus* venom is amongst the most potently coagulotoxic of any we have tested, being on par with *Oxyuranus* and *Pseudonaja* venoms tested under identical conditions (unpublished results). At a 0.05 µg/mL concentration, *T. mossambicanus* venom clots the plasma on average in 80.4 s (SD 0.52 s), compared to *D. typus*, which clots the plasma on average in 146.6 s (SD 5.23 s) (Table 1). Figure 6 demonstrates the relative potency of *T. mossambicanus* venom against human plasma, compared to *D. typus*, in the reduced variation in clotting times between dilutions. The distance between the first vertical line and the second vertical line is longer in *T. mossambicanus* than in *D. typus*, illustrating that there is a longer rise in clotting times, depicting that *T. mossambicanus* venom holds its potency between dilutions more so than *D. typus* (Figure 7, Table 2). This is also demonstrated by the point where the curve crosses the x axis, which is higher in *D. typus* than in *T. mossambicanus* (R0 *ϕ*_2_ = 37.83 and 23.17 s respectively) (Table 2). The additional investigation with and without cofactors CaCl_2_ and phospholipid had no impact on the clotting times of either species, with clotting times remaining the same at all dilutions (*data not shown*). This is indicative of both venoms being calcium and phospholipid independent in exerting their procoagulant coagulotoxic actions.

With the addition of diluted boomslang monovalent antivenom to the dilution series, it is evident that the antivenom neutralises *D. typus* remarkably well, in contrast to having little neutralising effect on *T. mossambicanus* (Figure 8). At 20 µg/mL venom concentration with the antivenom at a final concentration in the 250 µL cuvette volume of 1% of that of the original vial concentration, *T. mossambicanus* clotted the plasma in 9.6 s (SD 0.17 s) instead of the 7.5 s without antivenom, compared to *D. typus*, which clotted the plasma at 20.7 s (SD 1.07 s) instead of the 11.2 s without antivenom (Table 1). At a 0.05 µg/mL venom concentration, the added antivenom had some effect on *T. mossambicanus*, which clotted in 381.2 s (SD 4.71 s) instead of 80.4 s without antivenom (Table 1). *D. typus* however reached maximum clotting time of 999.9 s (Table 1) at a venom concentration of 0.05 µg/mL with the addition of antivenom, demonstrating a significantly greater effective neutralising ability against this species. 

**Figure 9:**
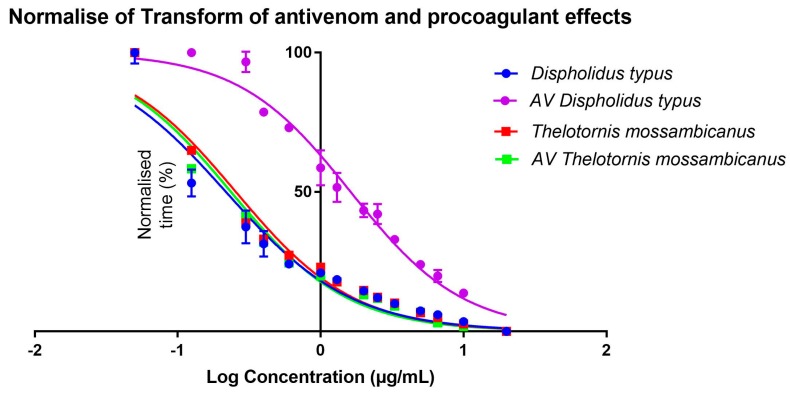
Normalisation of transformed data; antivenom and procoagulant effects of *D. typus* and *T. mossambicanus*. *D. typus* clotting curve given in blue circles, AV *D. typus* = antivenom response curve given in purple circles, *T. mossambicanus* clotting curve given in red squares, AV *T. mossambicanus* = antivenom response curve given in green squares. X axis: log concentration, Y axis: Normalised time (%) (for sub data sets). Analysis performed in GraphPad PRISM 7.0. Values are averages of triplicates (single dilution measured three times), and standard deviation error bars are shown for each, although for most the error range is smaller than the line icon.

Comparing EC_50_ outputs, it is evident that, despite the variation in clotting times between the species (Figure 8), half-maximal concentration is reached at a similar point by both species. This is due to the dilution series following the same trajectory regardless of time (Figure 8). When transformed, this trajectory becomes more evident, as there is little x-axis difference between *T. mossambicanus* with and without antivenom (0.24 µg/mL: 95% CI 0.22–0.26 µg/mL, and 0.23 µg/mL: 95% CI 0.20–0.26 µg/mL respectively) and *T. mossambicanus* and *D. typus* without antivenom (0.24 µg/mL and 0.20 µg/mL: 95% CI 0.18–0.23 µg/mL respectively) (Figure 9). However, when antivenom is introduced with *D. typus*, the EC_50_ x-axis shifts significantly to the right (1.62 µg/mL: 95% CI 1.49–1.76 µg/mL) (Figure 9). Due to each sub data set being transformed, first by log concentration and then the normalisation of clotting time, each final data point reaches 100% (Figure 9). Taking into account the y-axis shift in addition to the x-axis shift (Figure 8), the *T. mossambicanus* venom has an antivenom induced relative shift in the clotting curve of 4.69, while the *D. typus* venom has an antivenom induced relative shift in the clotting curve of 53.12. Thus the antivenom is 11.3 times more effective at neutralizing *D. typus* venom than *T. mossambicanus* venom. Thus if the antivenom had no significant effect upon *T. mossambicanus* under such ideal circumstances as conducted in this study, then there is little chance of it having an therapeutic effect in a clinical scenario without the use of extreme amounts of antivenom.

## 3. Discussion

Both venoms tested here were found to be highly procoagulant without requiring calcium or phospholipid as co-factors. However *T. mossambicanus* is notably more potent than *D. typus*, as well as being extremely poorly neutralised by SAIMR boomslang antivenom (Table 2, Figure 7, Figure 8 and Figure 9). The decoupling from co-factors is a notable discovery as this lack of shift between tests with or without co-factors had only been well-documented for *Echis carinatus*. This is the first extensive antivenom comparison of both species marking the relative effectiveness of the available boomslang monovalent antivenom.

Previous reports have described these two species as exhibiting strikingly similar lethal envenomations [17,19,20,21,30,37,38,39,40] that can be attributed to their venoms being dominated by P-III SVMPs (Figure 2 and Figure 3). It is notable that, despite *T. mossambicanus* having a lower concentration of SVMP due to its greater venom complexity, it displayed a higher relative rate of metalloprotease activity in addition to being more potently coagulotoxic (Figure 4, Figure 7 and Figure 8). Further, *T. mossambicanus* possesses two distinct molecular weight classes of SVMP as opposed to the single band present in *D. typus* venom (Figure 2). Thus the differential proteomics profile is mirrored by differential coagulotoxic activity. This key difference between the species’ venom profiles may also shed light as to why the available antivenom for *D. typus* has only a small neutralising effect on *T. mossambicanus* venom (Figure 8 and Figure 9, Table 1). 

Calcium-independent prothrombin activation is a rare feature in snake venoms, with most venoms typically requiring calcium for such a coagulotoxic activity. A previously documented notable exception is the ecarin-type P-III SVMP from *Echis carinatus*, which does not have a marked shift between tests with or without co-factors [25,45]. Thus, the presence of such an activity in the venoms of these two colubrid snakes points towards a remarkable case of functional convergence within the same toxin class.

Procoagulation accompanied by plasmin inhibition is an effective way to potentiate the coagulotoxic effects as the blood clots formed would have a longer half-life since the role of plasmin is to break blood clots down. This synergistic activity has been documented in viper venoms (e.g., *Daboia*) and elapid venoms (e.g., *Pseudonaja*), where the procoagulation is accomplished by very different toxin types (SVMP and fXa:fVa, respectively), but the plasmin inhibition is exerted by the same toxin type (kunitz peptide) [46,47]. However, neither *D. typus* or *T. mossambicanus* venoms displayed this synergistic action. 

Despite having the same procoagulant mechanism, it appears evident that additional external evolutionary pressures are driving the variation between these two species. In addition to differential complexity, the venoms were functionally variable relative to each other in non-procoagulant activities, with *D. typus* having serine protease activity (Figure 5), while conversely *T. mossambicanus* had strong PLA_2_ activity (Figure 6). It is known that evolution among arboreal specialists further influences venom composition of toxins’ families [48]. This is usually in the form of prey ecology, dependent on niche occupation. Even though both species occupy an arboreal habitat, their differential morphology and behaviour allow for varying prey interactions, thus potentially further contributing to venom variation and punctuated evolution. As *D. typus* is an agile and fast-moving snake, it is able to pursue prey, thus minimising escape potential. *T. mossambicanus* however is a slower moving cryptic snake that ambush feeds, and thus the prey escape potential may be significantly higher. Thus, there may be a stronger selection pressure operating upon *T. mossambicanus* for a venom which rapidly immobilises prey. This is consistent with what has been observed in other lineages of venomous animals in which prey escape potential is a significant shaping factor and thus the venom is under extreme selection pressure for rapid immobilising action [2,5,6,15,49,50,51,52,53,54,55,56,57,58,59]. 

This investigation not only has revealed the differential evolution of *D. typus* and *T.* *mossambicanus* venoms and the poor performance of boomslang antivenom against *T.* *mossambicanus* venom, but it has also reinforced that there are commonalities to venom evolution such that there may be levels of predictability in regards to niche occupation and prey escape potential shaping venom relative rates of action. The differences in ecological niches occupied were also reflected in differential skull morphology (Figure 1). This reinforces that, in addition to the applied application of venom research in regards to biodiscovery and clinical effects, research such as this contributes to the growing body of venom evolutionary theory.

## 4. Materials and Methods

### 4.1. Venom Supplies

Pooled venoms from *Dispholidus typus* (South African origin) and *Thelotornis mossambicanus* (Mozambique) were supplied by Latoxan (Portes-lès-Valence, France).

### 4.2. Micro-Computed Tomography (CT)

We scanned representative boomslang (*Dispholidus typus*) and the twig snakes (*Thelotornis mossambicanus*) with a Siemens Inveon micro-CT scanner. The scanner was operated at 80 KV energy, 250 µA intensity with 360 projections per 360°, and 2300 ms exposure time. The samples were scanned at a nominal isotropic resolution of 27.8 um. The data were reconstructed using a Feldkamp conebeam back-projection algorithm provided by an Inveon Acquisition Workstation from Siemens. The images in 3D were visualized and processed with ImageJ v1.51f [60], Materialise Mimics v19.0 (Materialise, Leuven, Belgium), and MeshLab v1.3.3 (Institute of the National Research Council of Italy, Pisa, Italy).

### 4.3. Magnetic Resonance Imaging

MRI was used to obtain a three-dimensional (3D) shape of the venom glands without intrusive dissection or sectioning techniques. For fixation, neutral buffered formalin (NBF) preserved specimens had the formalin removed by four individual hours of washing steps in phosphate buffered saline (PBS) and incubated overnight in 0.1% Magnevist^®^ (Bayer, Leverkusen, Germany) in PBS. After the removal of NBF, the sample was submersed in perfluoro-ether Fomblin (Solvay Solexis, Alessandria, Italy) and placed under vacuum to prevent air artifacts. Imaging was performed on a 16.4 T (700 MHz) vertical 89-mm-bore system (Bruker BioSpin, Rheinstetten, Germany) using a Bruker Micro 2.5 gradient system (2.5 G/cm A), transmit/receive radiofrequency coils with diameters of 10 mm, and a quadrature birdcage resonator (M2M Imaging, Brisbane, Australia). Bruker ParaVision 6.0.1 software was used for image acquisition and anatomical images were acquired using a 3D FLASH (Fast Low Angle Shot) gradient echo sequence. The imaging parameters were: TR/TE = 50/8 ms, flip angle 30 °C, two excitations. The field-of-view and matrix sized to fit the sample with the resulting voxels having 50–62.5 μm isotropic resolution. Total scan time was approximately 1 h per sample. MRI data was processed using Medical Imaging Processing, Analysis, and Visualization v6.0.0 (MIPAV, Centre for Information Technology, National Institutes of Health, Bethesda, MD, USA), and 3D image segmentation, surface rendering, and volumetric measurements of the glands were performed manually using ITK-SNAP [61].

### 4.4. Proteomics

Our proteomic investigations including using 1D and 2D SDS-PAGE, protein band purification and crude venom Shotgun analysis. They were performed as previously described by us [48] with the exception of additional LC-MS/MS analysis, following protein extraction from isolated 1D gel bands (described below in Section 4.4.1 and Section 4.4.2). Shotgun samples were analyzed using the methods described below (Section 4.4.3).

#### 4.4.1. Nano HPLC-ESI-Triple Time of Flight (TOF) Mass Spectral Analysis

Protein extracts from gel bands were analysed by LC-MS/MS using a Q Exactive™ Hybrid Quadrupole-Orbitrap Mass Spectrometer (Thermo Fisher Scientific, Waltham, MA, USA) equipped with a nano electrospray ion source. An aliquot (8 μL) of each extract was injected onto a C18 trap column (75 μm × 2 cm, Thermo Scientific, Waltham, MA, USA) at 6 µL/min. Samples were de-salted on the trap column for 5 min using 0.1% formic acid at 6 µL/min. The trap column was then placed in-line with the analytical nano HPLC column (15 cm × 75 μm C18, 3.5 μm, Thermo Fisher Scientific, Waltham, MA, USA) for mass spectrometry analysis. Solvent A consisted of 2% ACN/0.1% formic acid, and solvent B contained 100% ACN, 2% LC-MS water, and 0.1% formic acid. Peptide elution was made possible via linear gradients of 1 to 40% solvent B over 32 min at 300 nL/min flow rate, followed by a steeper gradient from 40 to 95% solvent B over 3 min. Solvent B was held at 95% for 2.5 min to allow for washing of the column and returned to 1% solvent B for equilibration prior to the next sample injection. The ion spray voltage was set to 1.8 kV, and the temperature of capillary was 300 °C. The mass spectrometer acquired 3 × 10^6^ ion count with the max injection time of 20 ms for a full scan TOF-MS data followed by 10^6^ ions count with the max injection time of 110 ms for a full scan product ion data in an Information Dependant Acquisition (IDA) mode. Full scan TOF-MS data was acquired over the mass range of 300–1800 m/z, while the product ion ms/ms was 100–1800 m/z. Ions observed in the TOF-MS scan exceeding a threshold of 1.8 × 10^5^ for the precursor selection with a charge state of ^+^1 to ^+^8 were set to trigger the acquisition of the product ion, with ms/ms spectra of the resultant 20 most intense ions. The data was acquired and processed using Xcalibur™ software (Thermo Fisher Scientific, Waltham, MA, USA).

#### 4.4.2. Protein Identification

Protein database searches were conducted against Uniprot for broad protein identification. A composite target decoy database was built with the forward and reverse sequences for calculating the FDR. Proteins were identified by database searching using PEAKS v7.5 (Bioinformatics Solutions Inc., Waterloo, ON, Canada) against the protein database. The search parameters were as follows: precursor ion mass tolerance, 10 ppm; fragment ion mass tolerance, 0.05 Da; fully tryptic enzyme specificity; one missed cleavage; monoisotopic precursor mass a fixed modification of cysteine carbamidomethylation; and variable modifications, including methionine oxidation, conversion of glutamine and glutamic acid to pyroglutamic acid, deamidation of asparagine, phosphorylation, acetylation, and sulfation. For PEAKS, *de novo* sequencing, database search, and characterising unspecific post-translational modifications (PTMs) were used to maximise the identifications; false discovery rate (FDR) was set to ≤1%; and the individual peptide ion score [−10·Log(p)] was calculated accordingly, where p is the probability that the observed match is a random event.

#### 4.4.3. Orbitap Elite Mass Spectrometer for SHOTGUNS

For LC-MS/MS analysis, the parameters are as follows; the samples were separated using RP-chromatography on a Dionex Ultimate 3000 RSLC nano-system (Lifetech, Carlsbad, CA, USA). The samples were desalted on a Thermo PepMap 100 C18 trap (Lifetech, Carlsbad, CA, USA) (0.3 × 5 mm, 5 µm) for 5 min with a flow rate of 30 µL/min. This was followed by separation on an Acclaim PepMap RSLC C18 (Lifetech, Carlsbad, CA, USA) (150 mm × 75 µm) column at a flow rate of 300 nL/min. A gradient of 10–70% was applied buffer B over 7 min, where buffer A (1% ACN/0.1% FA) and buffer B (80% ACN/0.1% FA) were used to separate peptides. The eluted peptides were directly analysed on an Orbitap Elite mass spectrometer (Thermo Scientific, Carlsbad, CA, USA) using an NSI electrospray interface. The source parameters included a capillary temperature of 275 °C; S-Lens RF level at 60%, source voltage of 2 kV, and maximum injection times of 200 ms for MS and 150 ms for MS2. The instrument parameters included an FTMS scan across m/z range 350–1800 at 60,000 resolution followed by information dependent acquisition of the top 10 peptides across m/z 40–1800. Dynamic ion exclusion was employed using a 15 s interval. Charge state screening was enabled with the rejection of +1 charged ions, and monoisotopic was precursor selection enabled. Data was converted to mascot generic format (mgf) using the msConvert software (ProteoWizard v3.0.9576, SCIEX, Concord, ON, Canada) and searched using Protein Pilot™ v5.0 (SCIEX, Concord, ON, Canada) via the Uniprot database “metazoan”.

### 4.5. Enzymatic Activity Assays

#### 4.5.1. Fluorescent Determination of Matrix Metalloprotease and Kallikrein Activity

A working stock solution of freeze dried venom was reconstituted in a buffer containing 50% MilliQ/50% glycerol (>99.9%, Sigma, St. Louis, MO, USA) at a 1:1 ratio to preserve enzymatic activity and reduce enzyme degradation. Varying concentrations of crude venom (10 ng/µL and 50 ng/µL) were plated out in triplicates on a 384-well plate (black, Lot#1171125, nunc™ Thermo Scientific, Rochester, NY, USA) and measured by adding 90 µL quenched fluorescent substrate per well (total volume 100 µL/well, 10 µL/5mL enzyme buffer, 150 mM NaCl, and 50 mM Tri-HCl (pH 7.3), Fluorogenic Peptide Substrate, R&D systems, Cat#ES001 & ES011, Minneapolis, MI, USA). Fluorescence was monitored by a Fluoroskan Ascent™ (Thermo Scientific, Vantaa, Finland) Microplate Fluorometer (Cat#1506450, Thermo Scientific, Vantaa, Finland) (Cat#ES001 for Matrix Metalloprotease at an excitation of 320 nm, emission at 405 nm; Cat#ES011 for Kallikrein at an excitation of 390 nm, emission at 460 nm) over 400 mins or until activity had ceased. Data was collected using Ascent^®^ Software v2.6 (Thermo Scientific, Vantaa, Finland).

#### 4.5.2. Fluorescent Determination of PLA_2_ Activity

We assessed the continuous Phospholipase A_2_ (PLA_2_) activity of the venoms using a fluorescence substrate assay (EnzChek^®^ Phospholipase A_2_ Assay Kit, Cat#E10217, Thermo Scientific, Rochester, NY, USA), measured on a Fluoroskan Ascent^®^ Microplate Fluorometer (Cat#1506450, Thermo Scientific, Vantaa, Finland). As above, we used a working stock solution of freeze dried venom reconstituted in a buffer containing 50% MilliQ/50% glycerol (>99.9%, Sigma) at a 1:1 ratio. A concentration of enzyme activity in venom (50 ng/µL) was brought up in 12.5 µL 1× PLA_2_ reaction buffer (50 mM Tris-HCL, 100 mM NaCl, 1 mM CaCl_2_, pH 8.9) and plated out in triplicates on a 384-well plate (black, Lot#1171125, nunc™ Thermo Scientific, Rochester, NY, USA). The triplicates were measured by dispensing 12.5 µL quenched 1 mM EnzChek^®^ (Thermo Scientific, Rochester, NY, USA) Phospholipase A_2_ Substrate per well (total volume 25 µL/well) over 100 min or until activity had ceased (at an excitation of 485 nm, emission 538 nm). Purified PLA_2_ from bee venom (1 U/mL) was used as a positive control and data was collected using Ascent^®^ Software v2.6 (Thermo Scientific, Vantaa, Finland).

#### 4.5.3. Enzymatic Statistical Analysis

For each of the methods performed on the Fluoroskan Ascent™ (Thermo Scientific, Vantaa, Finland) Microplate Fluorometer (Cat#1506450, Thermo Scientific, Vantaa, Finland), data was collected using Ascent^®^ Software v2.6 (Thermo Scientific, Vantaa, Finland). All raw data were firstly blank corrected using the Ascent^®^ software (v2.6, Thermo Scientific, Vantaa, Finland) package and then exported for further analysis using Windows Excel 2016 and GraphPad PRISM 7.0 (GraphPad Prism Inc., La Jolla, CA, USA).

Graphs: Using Excel, averages of blank corrected data triplicates were calculated. From these averages, maximum absorbance was calculated and absorbance value plotted in a column graph for relative percentages. 

Two-way (RM) ANOVA and Tukey’s Post-hoc test: the blank corrected data was transformed, setting the top value as 100 and lowest as 0 across sub columns within a data set. A two-way repeated measures (RM) ANOVA was performed, followed by a Tukey’s post-hoc multiple comparison test, comparing every mean with every other mean.

### 4.6. Procoagulation Analysis

#### 4.6.1. Whole Plasma Clotting

Healthy human plasma (citrate 3.2%, Lot#1690252, approval # 16-04QLD-10) was obtained from the Australian Red Cross (44 Musk Street, Kelvin Grove, Queensland 4059). Coagulopathic toxin effects were measured by a modified procoagulant protocol on a Stago STA-R Max coagulation robot (France) using Stago Analyser software v0.00.04 (Stago, Asniéres sur Seine, France). Plasma clotting baseline parameters were determined by performing the standardised activated Partial Thromboplastin Time (aPTT) test (Stago Cat# T1203 TriniCLOT APTT HS). This was used as a control to determine the health of normal clotting plasma according to the universal standard range of between 27–35 s. Plasma aliquots of 2 mL, which had been flash frozen in liquid nitrogen and stored in a −80 °C freezer, were defrosted in an Arctic refrigerated circulator SC150-A40 at 37 °C. In order to determine clotting times effected by the addition of varying venom concentrations, a modified aPTT test was developed. A starting volume of 50 µL of crude venom (5 µg/50 µL) was diluted with STA Owren Koller Buffer (Stago Cat# 00360). A 14-dilution series of 1 (20 µg/mL), 1/2 (10 µg/mL), 1/3 (6.66 µg/mL), 1/4 (5 µg/mL), 1/6 (3.33 µg/mL), 1/8 (2.5 µg/mL), 1/10 (2 µg/mL), 1/15 (1.33 µg/mL), 1/20 (1 µg/mL), 1/30 (0.66 µg/mL), 1/50 (0.4 µg/mL), 1/80 (0.25 µg/mL), 1/160 (0.125 µg/mL), and 1/400 (0.05 µg/mL) was performed in triplicate. CaCl_2_ (50 µL; 25 mM stock solution Stago Cat# 00367 STA CaCl_2_ 0.025M) was added with 50 µL phospholipid (solubilized in Owren Koller Buffer adapted from STA C.K Prest standard kit, Stago Cat# 00597). An additional 25 µL of Owren Koller Buffer was added to the cuvette and incubated for 30 s at 37 °C before adding 75 µL of human plasma. Relative clotting was then monitored for 999 s or until plasma clotted (whichever was sooner). Additional tests were run, both with and without CaCl_2_ and phospholipid, respectively, to test for CaCl_2_ or phospholipid dependency. STA Owren Koller Buffer was used as a substitute, using the same volumes to allow for consistency in the final volumes (250 µL).

#### 4.6.2. Antivenom Studies

The monovalent antivenom effects on both *D. typus* and *T. mossambicanus* crude venoms was investigated. Previously established whole plasma clotting times against both venoms were measured and used as a guide for antivenom effects. The South African Institute for Medical Research Boomslang Antivenom (refined equine immunoglobulins, Lot M03852) was purchased from South African Vaccine Producers Pty Ltd (1 Modderfontein Rd Edenvale, Gauteng, South Africa). One vial of antivenom (10 mL) was centrifuged at 12,000 rpm on an Allegra™ X-22R Centrifuge (Lot#982501, Beckman Coulter, Brea, CA, USA) for 10 min at 4 °C, supernatant extracted, filtered (0.45 µm Econofltr PES, Lot#131127028, Agilent Technologies, Beijing, China) and stored at 4 °C. A final stock solution of 10% antivenom and 90% Owren Koller Buffer was produced. Modifying the Section 4.4.1 protocol, 25 µL of antivenom stock was used in place of 25 µL of OK buffer, with the venom, Ca^2+^, phospholipid, and antivenom mixture being incubated for 120 s at 37 °C before adding 75 µL of human plasma and monitoring clotting for 999 s or until plasma clotted (whichever was sooner). Experiments were conducted in triplicate. Note that antivenom does not clot plasma and that a control was performed to rule out any additional effects antivenom has on the plasma. Antivenom was substituted into the above outlined protocol in replacement of a venom sample.

#### 4.6.3. Statistical Analysis

##### Whole Plasma Clotting EC_50_ Concentration and Asymptotic Time

Venom dilutions in triplicates were mapped over time using GraphPad PRISM 7.0 to produce concentration curves. The statistical program R [62] was used to calculate asymptotic clotting times using the Asymptotic Regression Model. This can be written as *y*(*x*) = *ϕ*_1_ + (*ϕ*_2_ − *ϕ*_1_) exp[−exp(*ϕ*_3_)*x*], using the nlme package [63]. This asymptotic regression model was used to model the response of *y* (time) when approaching a horizontal asymptote, when *x* (concentration) approaches ∞ [64]. In this example, *ϕ*_1_ is the asymptote as *x* (concentration) approaches ∞, *ϕ*_2_ is the response at *x* = 0, and *t*_0.5_ is the half-life. Parameter *ϕ*_3_ is the logarithm of the rate constant that is used to enforce positivity so that an asymptote is reached in the model. The corresponding half-life is written as *t*_0.5_ = log2/exp(*ϕ*_3_). The EC_50_ can be explained as the half-maximal Effective Concentration, or the concentration at which 50% of the maximal effect is observed. To calculate this, the EC_50_ function in GraphPad PRISM 7.0 was used for each data set. Each data set was transformed by the log of the concentration, and the values were normalised by individual sub data sets. A non-linear regression curve fit was then modelled, and a log-reversed EC_50_ value was produced.

##### Antivenom EC_50_ Concentration

Venom dilutions in triplicates were mapped over time using GraphPad PRISM 7.0 to produce concentration curves. The EC_50_ function in GraphPad PRISM 7.0 was used to calculate the EC_50_ for each data set. Each data set was transformed by the log of the concentration, and the values were normalised by individual sub data sets. A non-linear regression curve fit was then modelled, and a log-reversed EC_50_ value was produced.

## Figures and Tables

**Table 1:** Average clotting times with standard deviations (SD) (in seconds) of *D. typus* and *T. mossambicanus* at varying dilutions with and without the addition of boomslang antivenom.

Concentration	Without Antivenom				With Antivenom			
	*D. typus*		*T. mossambicanus*		*D. typus*		*T. mossambicanus*	
	Mean	*SD*	Mean	*SD*	Mean	*SD*	Mean	*SD*
20 µg/mL	11.27	*0.06*	7.57	*0.15*	20.73	*1.07*	9.6	*0.17*
10 µg/mL	15.95	*1.88*	9.55	*0.17*	155	*5.2*	16	*0.17*
6.66 µg/mL	19.34	*0.21*	11.20	*0.34*	214.56	*21.28*	21.16	*0.23*
5 µg/mL	21.22	*0.22*	12.43	*0.08*	255	*10.56*	35.13	*1.32*
3.33 µg/mL	24.72	*0.22*	14.95	*0.65*	343.2	*8.97*	43.33	*1.86*
2.5 µg/mL	27.63	*0.14*	16.45	*0.18*	432.3	*35.32*	53.43	*0.11*
2 µg/mL	30.91	*0.47*	18.27	*0.13*	444.93	*23.43*	58.46	*1.29*
1.33 µg/mL	36.32	*1.61*	20.50	*0.58*	525.86	*51.19*	75.43	*2.15*
1 µg/mL	39.60	*0.54*	24.30	*0.74*	594.86	*61.15*	83.33	*3.86*
0.66 µg/mL	43.91	*0.56*	27.44	*1.03*	735.3	*6.94*	100.8	*1.97*
0.4 µg/mL	53.77	*6.21*	31.67	*0.19*	790.36	*9.41*	128.4	*0.52*
0.25 µg/mL	61.95	*7.95*	35.91	*0.64*	966.76	*35.88*	163.23	*2.6*
0.125 µg/mL	83.31	*6.51*	54.89	*0.14*	999	*0*	226.33	*2.6*
0.05 µg/mL	146.65	*5.23*	80.44	*0.52*	999	*0*	381.23	*4.71*

**Table 2:** Asymptotic regression model coefficient estimates for *D. typus* and *T. mossambicanus* normal plasma clotting times. In this example, *ϕ*_1_ is the asymptote as *x* (concentration) approaches ∞, *ϕ*_2_ is the response at *x* = 0, and *t*_0.5_ is the half-life. Parameter *ϕ*_3_ is the logarithm of the rate constant which is used to enforce positivity so that an asymptote is reached in the model. The corresponding half-life is written *t*_0.5_ = log2/exp(*ϕ*_3_). Analysis and output performed in R Studio (refer to methodology 4.4.3 Coagulation Statistical analysis).

Species	Coefficient Estimates		
	Aym *ϕ*_1_	R0 *ϕ*_2_	lrc *ϕ*_3_
*D. typus*	9.095161 (s)	37.83835 (s)	−0.6711807
*T. mossambicanus*	2.068652 (s)	23.17609 (s)	−0.8289048

## Supplementary Materials

The following are available online at www.mdpi.com/2072-6651/9/5/171/s1, Supplementary data: MS/MS data.

## Abbreviations

The following abbreviations are used in this manuscript:

CI	confidence interval
aPTT	activated partial thromboplastin time
SVMP	snake venom metalloprotease
SDS-PAGE	sodium dodecyl sulfate polyacrylamide gel electrophoresis
CRiSP	cysteine rich secretory protein
HPLC	high pressure liquid chromatography
SD	standard deviations
ACN	acetonitrile
1D	one dimensional
2D	two dimensional
3FTx	three finger toxin
PLA_2_	phospholipase A_2_
